# Supplementary material for: Flexible Stretchable, Dry-Resistant MXene Nanocomposite Conductive Hydrogel for Human Motion Monitoring
Source: Polymers (Basel). 2023 Jan 4;15(2):250. doi: 10.3390/polym15020250 (PMC9864371; doi:10.3390/polym15020250)
Supplement: Supplementary file 1 [file polymers-15-00250-s001.zip › polymers-2110141-supplementary.pdf]

2. Materials and Methods

Electrochemical performance: The hydrogel was cut into regular pieces (20 mm in length, 10 mm in width, and 8 mm in height), connected with copper wires on both sides, connected to the electrochemical workstation, and set a constant voltage of 5 mV. The hydrogel was applied to the index finger joint, and when the finger was bent at different angles, the resistance value of the hydrogel changed, to determine the ability of the hydrogel to convert pressure signals into electrical signals. The rate of change of relative resistance is defined as:

$$\Delta R(\%) = \frac{(R - R_0)}{R_0} \times 100\%$$

where R and R_0 were the immediate resistance value and the initial resistance value of the hydrogel when strain is generated in Ω, respectively.

The strain factor (GF) is defined as

$$GF = \frac{(R - R_0)/R_0}{\varepsilon}$$

where ε represents the strain.

3. Results

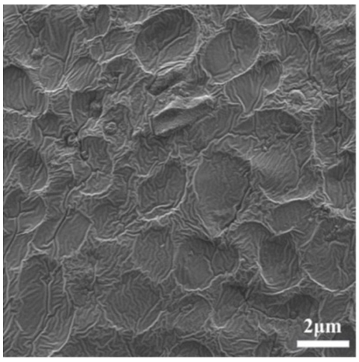

Figure S1. SEM image of a fish-scale hydrogel.

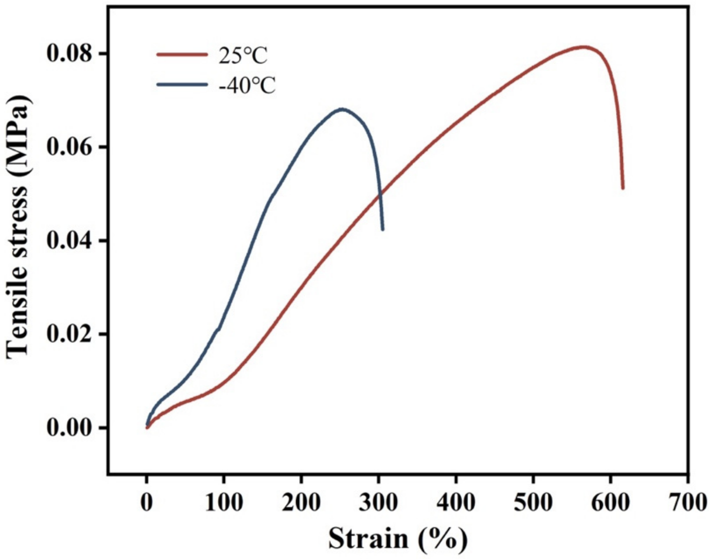

Figure S2. Comparison of the tensile properties of PAEM at room temperature and at -40 °C.
